# Variable Resistance to Plasminogen Activator Initiated Fibrinolysis for Intermediate-Risk Pulmonary Embolism

**DOI:** 10.1371/journal.pone.0148747

**Published:** 2016-02-11

**Authors:** William B. Stubblefield, Nathan J. Alves, Matthew T. Rondina, Jeffrey A. Kline

**Affiliations:** 1 Louisiana State University, Health Sciences Center, New Orleans, United States of America; 2 Indiana University School of Medicine, Indianapolis, United States of America; 3 University of Utah School of Medicine, Salt Lake City, United States of America; 4 Department of Emergency Medicine, and Department of Cellular and Integrative Physiology, Indiana University School of Medicine, Indianapolis, IN, United States of America; IIBB-CSIC-IDIBAPS, SPAIN

## Abstract

**Background:**

We examine the clinical significance and biomarkers of tissue plasminogen activator (tPA)-catalyzed clot lysis time (CLT) in patients with intermediate-risk pulmonary embolism (PE).

**Methods:**

Platelet-poor, citrated plasma was obtained from patients with PE. Healthy age- and sex-matched patients served as disease-negative controls. Fibrinogen, α_2_-antiplasmin, plasminogen, thrombin activatable fibrinolysis inhibitor (TAFI), plasminogen activator Inhibitor 1 (PAI-1), thrombin time and D-dimer were quantified. Clotting was induced using CaCl_2_, tissue factor, and phospholipid. Lysis was induced using 60 ng/mL tPA. Time to 50% clot lysis (CLT) was assessed by both thromboelastography (TEG) and turbidimetry (A405).

**Results:**

Compared with disease-negative controls, patients with PE exhibited significantly longer mean CLT on TEG (+2,580 seconds, 95% CI 1,380 to 3,720 sec). Patients with PE and a short CLT who were treated with tenecteplase had increased risk of bleeding, whereas those with long CLT had significantly worse exercise tolerance and psychometric testing for quality of life at 3 months. A multivariate stepwise removal regression model selected PAI-1 and TAFI as predictive biomarkers of CLT.

**Conclusion:**

The CLT from TEG predicted increased risk of bleeding and clinical failure with tenecteplase treatment for intermediate-risk PE. Plasmatic PAI-1 and TAFI were independent predictors of CLT.

## Introduction

Clinical trials have suggested that treating intermediate-risk, acute PE patients with fibrinolytics reduces hemodynamic complications but at a cost of increased bleeding risk [1–3]. In four meta-analyses by Chatterjee et al. Nakamura et al., Marti et al., and Riera-Maestre et al. the rates of major bleeding with fibrinolytic treatment for PE were 9.2%, 6.6%, 9.9%, and 5.9% respectively. Intracranial bleeding rates were 1.5%, 1.7%, 1.7%, and 1.7% respectively [4–7].

Experimental data in animals [8] and humans [9, 10] demonstrate that the bleeding rate increases with increasing dose of fibrinolytic agent. Recent work has been hypothesis generating in the use of half-dose tPA to reduce right ventricular dysfunction after PE compared with no fibrinolysis, while potentially decreasing the bleeding risk associated with full-dose tPA [11–13]. It can be hypothesized that the dose of plasminogen activator for PE should be tailored to patient phenotype including clot size, patient body weight, age, and gender, as well as circulating proteins that determine the degree of plasmatic resistance to tPA fibrinolysis. Our objectives were first to examine the frequency of plasmatic resistance to tPA in patients with intermediate-risk PE, and then to determine which plasma proteins have independent predictive value for determining susceptibility to tPA-catalyzed clot lysis. The overarching intent is to identify a biomarker, or biomarker panel, to predict the risk of hemorrhage or poor clinical response with standard dose fibrinolytics. To accomplish this, we used two commonly used methods for assessing clot lysis time (CLT): turbidimetry and thromboelastography (TEG) on plasma samples from patients with intermediate-risk PE [1]. We then assessed the predictiveness of CLT and correlated biomarkers on known hemorrhagic and clot-related, clinical outcomes.

## Methods

### Study design

Plasma from the primary study group was obtained from a prospective, multicenter trial for treatment of intermediate-risk pulmonary embolism (TOPCOAT), clinical trials identifier: NCT00680628 [1]. Carolinas HealthCare System Institutional Review Board approved the original study (IRB #01-08-01A) and this study had prospective ethical approval. The methods for TOPCOAT are detailed in a separate publication [14]. Patients were randomized to receive a bolus infusion of the plasminogen activator tenecteplase (TNKase®) or volume-matched 0.9% NaCl placebo. To provide disease-negative control data, we used plasma from patients who were matched to TOPCOAT by age and sex from apparently healthy individuals who were tested for acute PE, but had no clinical evidence of PE within 90 days. To provide positive control data, we used plasma from patients with conditions known to produce hypofibrinolysis via different mechanisms, including patients with diabetes mellitus type II, and patients with metabolic syndrome [15, 16]. All patients in this study provided written informed consent. Blood was obtained prior to treatment from an arm vein, either by venipuncture or withdrawal from an indwelling venous catheter, into a vacuum tube containing sodium citrate (BD Vacutainer®, 2.7 mL, 0.109 Molar/3.2% sodium citrate). Phlebotomy in TOPCOAT patients was performed prior to administration of study drug. Blood was immediately placed on ice, and centrifuged within 30 minutes at 4°C at 3,000 x g for 20 minutes, which has been shown to deplete platelets [17]. Plasma was immediately aliquoted and frozen at -80°C.

### Analysis of Clot Lysis Time

We used two methods to assess clot lysis time (CLT) to mimic two distinct in-vivo physiological conditions. First, to represent CLT in stagnant (zero shear) conditions, we measured turbidity using light transmittance (SpectraMax, Molecular Devices; Sunnyvale, CA). Second, to measure CLT under shear conditions, we assessed loss of mechanical stiffness, using TEG (Haemoscope 5000; Braintree, MA). For both assays, plasma comprised 50% of the total mixture volume. Turbidity was measured at 405nm at 37°C [18]. Coagulation was initiated by inducing clotting with calcium chloride at a final concentration of 15 mM, human tissue factor at a final concentration of 0.6 pM (Dade Innovin; Siemens, USA), and phospholipids, DOPC:DOPS (7:3, w/w), at a final concentration of 12 μM (Avanti Polar Lipids; Alabaster, Al) [18–20]. To induce fibrinolysis, tissue plasminogen activator (tPA) (Alteplase, Genentech; San Francisco, CA) was immediately added to the plasma prior to clot formation at a final concentration of 60 ng/mL. Tris-buffered saline (50 mM Tris-HCl, 0.1 M NaCl, pH 7.4) was used as a buffering agent. Calcium chloride, tissue factor, phospholipid mixture, tPA, and buffer were mixed in disposable TEG cups containing heparinase (Haemonetics Corporation; Braintree, MA) prior to addition of plasma. Following mixing of reagents, human plasma was added to start the reaction. 100 μL of volume was transferred from the TEG cup to a 96 well plate in duplicate, and allowed to run on the spectrophotometer at 23°C. The remaining reaction volume was run using TEG. The CLT is derived from a clot-lysis profile and defined as the time between the midpoint of the rise to maximum turbidity or shear stress during coagulation, to the midpoint of the return back to baseline turbidity or shear stress during clot lysis (S1A–S1D Fig).

### Measurement of plasma proteins

All plasma proteins with the exception of PAI-1 were measured on the STA Compact coagulation analyzer® (Diagnostica Stago; Parsippany, NJ) with reagents purchased from the manufacturer and analyzed as follows: Fibrinogen concentration (mg/dL) was determined using the Clauss clotting method (STA Fibrinogen 5), α_2_-antiplasmin, plasminogen and thrombin activatable fibrinolysis inhibitor (TAFI) concentrations were determined via chromogenic assays (STA Stachrom α_2_-antiplasmin, STA Stachrom plasminogen and STA Stachrom TAFI values expressed as a percent relative to the standard control). All assays were performed with the use of a commercial calibration standard; D-dimer concentrations (μg/mL) were measured using a latex agglutination assay (STA Liatest D-DI). Plasminogen activator Inhibitor 1 (PAI-1, pg/mL) was quantified with a commercial ELISA assay (Life Technologies, Grand Island, NY). The Life Sciences Human PAI-1 kit is a solid phase sandwich Enzyme Linked-Immuno-Sorbent Assay (ELISA). A monoclonal antibody specific for Hu PAI-1 has been coated onto the wells of the microtiter strips provided. The assay used in this study measures total PAI-1.

### Clinical outcomes

To assess the association of CLT with clinical outcomes from the TOPCOAT sample, we grouped patients according to CLT value. First, we assessed the area under the receiver operating characteristic curve, as well as the significance of a short CLT (below the 1^st^ quartile for disease-negative patients) on risk of major or clinically relevant non-major bleeding. Bleeding was defined as major if it was clinically overt and associated with a decrease in hemoglobin level of ≥2.0 g/dL; if bleeding led to the transfusion of ≥2 units of red cells; or if bleeding was intracranial or retroperitoneal, occurred in another critical site, or contributed to death [21]. Non-major clinically relevant bleeding was defined as overt bleeding that did not meet the criteria for major bleeding but was associated with medical intervention drug, or discomfort or impairment of activities of daily life. Second, we assessed the association of a prolonged CLT (CLT > 95th percentile from the disease-negative group) with five efficacy outcomes assessed at three months post treatment: 1. Baseline pulse oximetry, 2. Quality of life related to post-thrombotic syndrome as measured by the validated VEINES QoL survey [22] 3. Perception of physical and mental wellness from the RAND Standard Form 36 (SF 36) [23] 4. Six-minute walk distance (m), [24] and 5. The proportion of patients with evidence of right ventricular dysfunction or overload on echocardiography, defined as RV dilation (>43 mm transverse diameter in diastole), RV hypokinesis, or an estimated RV systolic pressure >45 mmHg.

### Statistical analysis

Samples were evaluated for normality using the Shapiro-Wilk test. Means were compared using analysis of variance with Dunnett’s post-hoc test to determine significance with pairwise comparisons of test groups (PE, diabetes mellitus and metabolic syndrome) versus control with p < 0.05 considered significant. To determine which variables explain the change in CLT in the PE samples, we performed multivariate linear regression with age, BMI, fibrinogen, D-dimer, plasminogen, α_2_-antiplasmin, thrombin time, TAFI and PAI-1 as independent variables and CLT from turbidimetry or TEG as the dependent variable using two separate equations. The stepwise removal process was then used to select significant independent variables. Data were analyzed using SPSS (Version 22; IBM, Armonk NY). Graphs were produced using SigmaPlot (version 12.0; Systat, San Jose, CA).

## Results

Table 1 compares clinical features between the three control groups and the experimental group: normal (n = 20, negative control), metabolic syndrome (n = 10, positive control), diabetes mellitus (n = 10, positive control), and intermediate-risk PE patients from TOPCOAT. Of these patients (n = 76 total), 36 were treated with tenecteplase, and 40 treated with placebo). All TOPCOAT patients received either full-dose unfractionated heparin (n = 34), low molecular weight heparin (n = 49), or both (n = 10) prior to blood draw. The four groups were similar in age, but patients with metabolic syndrome and TOPCOAT group had a higher body mass index (p < 0.05). Table 1 also compares relative concentrations and activities of biomarkers relevant to fibrinolysis in the three control groups and PE patients. As expected, D-dimer and fibrinogen concentrations were elevated in patients with PE, and thrombin times were prolonged from heparin treatment. PAI-1 was significantly increased in both the positive control groups and the PE group when compared with disease-negative controls. The mean plasma antigenic content of α_2_-antiplasmin, plasminogen, or TAFI were not different between groups.

**Table 1:** Comparison of clinical data and plasma proteins between patient groups.

Variable	Control (n = 20)	Diabetes Mellitus (n = 10)	Metabolic Syndrome (n = 10)	TOPCOAT (Intermediate-risk PE) (n = 76)
Age	56.5 ± 14.6	57.8 ± 14.2	65.1 ± 20	55 ± 13.9
Male gender (%)	11 (55%)	5 (50%)	7 (70%)	46 (61%)
Body mass index (kg/m^2^)	27.9 ± 8.1	31.3 ± 5.7	34.7 ± 4.8*	33.1 ± 9.1*
Diabetes Mellitus	0	10	0	10
Prior venous thromboembolism	0	0	0	17
Active malignancy	0	0	0	15
Thrombin time (s)	18.1 ± 2	19.6 ± 1.2	19.8 ± 1.7	61 ± 47.1*
Fibrinogen (mg/dL)	320.9 ± 54.7	350.7 ± 52	338 ± 70.7	412.5 ± 148.9*
α_2_-antiplasmin†	102.7 ± 12.3	103.2 ± 5.7	106.4 ± 6.7	99.2 ± 18.2
Plasminogen†	98.2 ± 16	102.3 ± 11.3	104.5 ± 13.7	109.8 ± 27.2
TAFI†	107.3 ± 16.9	104.3 ± 18.6	106.9 ± 18.7	99.5 ± 25.7
D-dimer (μg/mL)	0.447 ± 0.429	0.360 ± 0.141	0.429 ± 0.307	6.592 ± 5.102*
PAI-1 (pg/mL)	1072.3 ± 780.1	3457 ± 2518.7*	4171.3 ± 2177.7*	2367.5 ± 2212.9*

*p < 0.05 from one-way ANOVA with Dunnett’s comparison with control

†Units are expressed as percent activity when compared to standardized controls provided by the manufacturer (Stago Diagnostica). Abbreviations: TAFI-thrombin activatable fibrinolysis inhibitor; PAI-plasminogen activator inhibitor. Values are listed as mean ± SD unless otherwise indicated.

**Fig 1:**
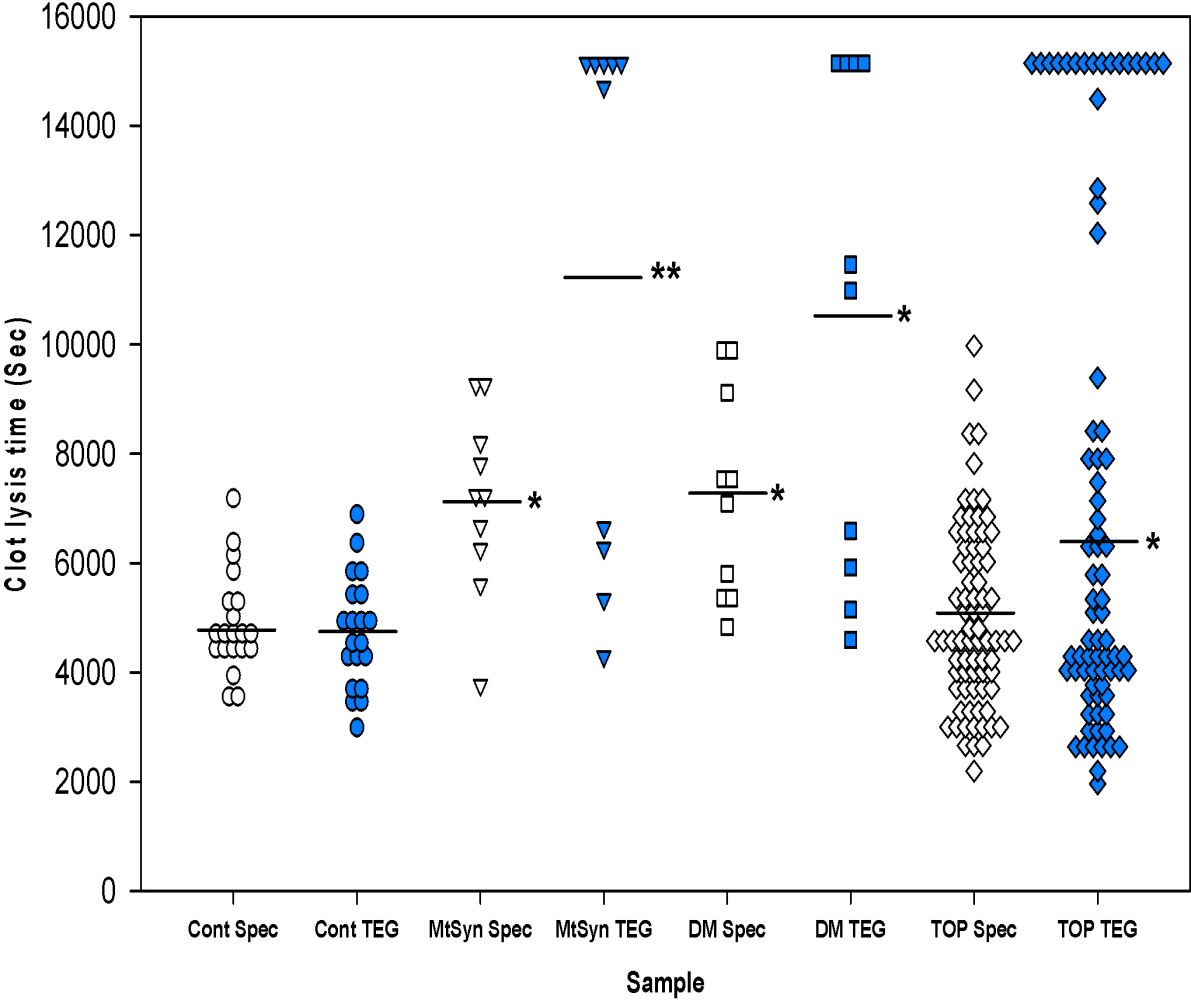
Dot plot of clot lysis time values for each patient. Horizontal lines represent the mean of each group. Abbreviations: Cont-apparently healthy control patients; TEG-thromboelastography; MtSyn- metabolic syndrome; DM-diabetes mellitus; Spec-spectrophotometry (turbidimetric method); TOP-TOPCOAT. *p < 0.05 vs. control, **p < 0.01 vs. control, ANOVA with Dunnett’s post-hoc.

Fig 1 and Table 2 show the CLTs measured with both turbidimetry and TEG (S1A–S1D Fig in the online supplement demonstrates the methodology for turbidimetry, and S2A and S2B Fig shows the regression curves and Bland Altman plots, respectively comparing the CLT results from each method). As measured by TEG, the mean CLT was significantly prolonged for patients with PE, diabetes mellitus and metabolic syndrome compared with controls (p = 0.03, p = 0.0026, and p = 0.0005, respectively). A significantly higher proportion of patients with PE (18%) had a CLT > 10,800 seconds compared with controls (0%) (95% confidence interval for the difference in 18% = 0.3 to 27%). Using the turbidimetric technique, the mean CLT was not significantly prolonged for patients with PE compared with disease-negative controls, but was prolonged in patients with diabetes mellitus and metabolic syndrome compared with controls (p = 0.623, p = 0.002 and p = 0.003, respectively).

**Table 2:** Clot lysis times (seconds) from thromboelastography and turbidimetry.

Value	Healthy Control (Spec)	Diabetes Mellitus (Spec)	Obese Metabolic Syndrome (Spec)	TOPCOAT (Spec)	Total Sample (Spec)	Healthy Control (TEG)	Diabetes Mellitus (TEG)	Obese Metabolic Syndrome (TEG)	TOPCOAT (TEG)	Total Sample (TEG)
Valid data (N)	20	10	10	76	116	20	10	10	76	116
Mean	4,898	7,243*	11,296*	5,014	5,369	4,741	10,529*	11,296*	7,319*	7,494
SD	920	1,907	4,914	1,657	1,770	1,029	4,561	4,914	4,735	4,659
Upper quartile	5,319	9,303	8,465	6,284	6,574	5,440	15,145	15,145	12,449	12,449
Median	4,685	7,308	7,235	4,628	5,018	4,703	11,225	14,925	5,095	5,363
Lower quartile	4,441	5,364	6,088	3,739	4,085	3,842	5,728	6,039	3,780	4,081
Centile 95	7,145	9,280	9,935	8,344	9,181	6,869	15,145	15,145	15,145	15,145
Centile 10	3,465	4,830	3,765	2,735	2,957	3,013	4,595	4,285	2,613	2,633

*p < 0.05 from one-way ANOVA with Dunnett’s comparison with control

Abbreviations: Spec-spectrophotometry (turbidimetric method); TEG-Thromboelastography; TOPCOAT-tenecteplase or placebo: cardiopulmonary outcomes at three months; SD-Standard deviation.

To determine significance of CLT on patients with major bleeding, we first tested if CLT (measured via TEG) predicted hemorrhage associated with tenecteplase treatment using receiver operating curve analysis. Of the 36 patients with PE who received tenecteplase, one had major hemorrhage (fatal intracranial hemorrhage), one had severe hemorrhage that did not meet the study definition of major hemorrhage (vaginal bleeding associated with hypotension and red cell transfusion, but no drop in hemoglobin), and six others that met the definition of clinically relevant non-major bleeding. Average age and BMI of these eight patients were 61.4 and 30.9, respectively, and all patients with hemorrhage had met criteria for TOPCOAT enrollment. The CLT was significantly lower in the eight patients with hemorrhage (3,823 ± 1,456 seconds) versus 28 patients without hemorrhage (6,761 ± 4,321 seconds, p = 0.005 unpaired t-test). The diagnostic accuracy of CLT from TEG in predicting major or clinically significant bleeding was moderate with an area under the ROC of 0.711 (95% CI = 0.526 to 0.892). The odds ratio for hemorrhage for patients with CLT < 1^st^ quartile of TOPCOAT patients (3,780 seconds) was 11.6 (95% CI = 1.6–104), and both patients with major or near major hemorrhage had a CLT below 3,780 seconds. Table 3 compares the mean values for five outcomes at three months for patients with a prolonged CLT (i.e., > 95 percentile for healthy controls or > 6,869 seconds) and found significantly lower VEINEs QoL, physical component of the SF-36 and the six-minute walk distance values in patients treated with tenecteplase. Additionally, we assessed the percentage of patients with right ventricular (RV) dysfunction or overload at 3 months. Of the 36 patients treated with tenectaplase, there were 12 patients with prolonged CLT. For those treated with tenecteplase, RV dysfunction or overload was found in 36% with prolonged CLT, versus 26% with normal CLT (95% CI for difference, -20.6 to 42.4%, exact two-sided p = 0.46), and for placebo, RV dysfunction or overload was found in 54% with prolonged CLT, versus 27% with normal CLT (95% CI for difference of 17% = -5.1 to 55.4%, exact two-sided p = 0.095).

**Table 3:** Clinical outcomes three months after pulmonary embolism based upon clot lysis time at diagnosis.

Clinical parameter	Tenecteplase, normal CLT	Tenecteplase, prolonged CLT*	P‡	Placebo, normal CLT	Placebo, prolonged CLT*	P‡
Resting pulse oximetry value (%)	97.0 ± 1.4	96.5 ± 1.8	0.400	97.0 ± 1.7	97.0 ± 1.3	0.931
Lowest recorded pulse oximetry value (%)	92.9 ± 5.5	86.3 ± 14.3	0.057	93.1 ± 3.6	92.6 ± 4.3	0.704
VEINEs QoL score**	96.4 ± 10.5	85.9 ± 12.1	0.013	86.5 ± 19.4	93.5 ± 13.4	0.263
Physical component of the SF-36** (Normalized values at 3 month follow up)	49.8 ± 7.9	42.0 ± 10.3	0.019	41.2 ± 12.8	41.5 ± 14.1	0.952
Mental component of the SF-36 (Normalized values at 3 month follow up)	52.6 ± 8.1	54.1 ± 11.6	0.674	51.6 ± 12.3	55.2 ± 4.7	0.323
Six-minute walk distance (feet at 3 month follow-up)	1472.9 ± 261.1	1094.1 ± 328.6	0.002	1338.0 ± 423.5	1350.4 ± 318.4	0.931

Values are listed as Mean±SD

Abbreviations: CLT-clot lysis time; OoL-quality of life

*Prolonged CLT defined as > 95th percentile using thromboelastography.

**Increasing values indicate better quality of life.

‡Unpaired t-test, comparing patients with normal CLT to patients with prolonged CLT.

We then sought to determine which biomarkers correlated to CLT using multivariate linear regression for each technique, using data from all 116 patients (TOPCOAT + normal + metabolic syndrome + diabetes mellitus). Table 4 presents the β coefficients and p values for each variable entered. Examination of the β coefficients and associated p values suggest PAI-1 as the most robust predictor of CLT in both the turbidimetry and TEG methods. For the turbidimetry regression model, stepwise variable removal retained biomarkers D-dimer, PAI-1 and α_2_-antiplasmin, yielding a best-fit prediction model with F (3, 113) = 36.3, p < 0.001, that explained 49.3% of the variance of CLT by turbidimetry (r^2^ = 0.493). For the TEG regression model, stepwise variable removal retained biomarkers PAI-1 and TAFI, yielding a significant best-fit prediction model, F (3, 113) = 61.2, p < 0.001, that explained 62.1% of the variance of CLT by TEG (r^2^ = 0.621). Neither equation produced clinically useful prediction for hemorrhage or the outcomes in Table 3.

**Table 4:** β coefficients and P values for independent variables of multivariate linear regression*.

	Turbidimetry		Thromboelastography	
Component	Beta coefficient	P value	Beta coefficient	P value
Intercept	b0 = 1,279.8	P = 0.31	b0 = -3,232.5	P = 0.261
Age	b1 = 5.107	P = 0.556	b1 = 0.55	P = 0.978
BMI	b2 = 12.94	P = 0.388	b2 = 40.18	P = 0.241
Fibrinogen	b3 = 0.639	P = 0.6	b3 = 0.74	P = 0.79
D-Dimer	b4 = -61.07	P = 0.031	b4 = 37.46	P = 0.558
Plasminogen	b5 = 1.56	P = 0.836	b5 = -2.70	P = 0.876
α_2_-antiplasmin	b6 = 15.67	P = 0.108	b6 = 31.34	P = 0.158
Thrombin time	b7 = -2.59	P = 0.415	b7 = -9.14	P = 0.208
TAFI	b8 = 5.94	P = 0.373	b8 = 25.65	P = 0.093
PAI-1	b9 = 0.49	P < 0.001	b9 = 1.62	P < 0.001
df = 115, F = 12.24 P < 0.001, Ra^2^ = 46.8%			df = 115, F = 20.218 P < 0.001, Ra^2^ = 60.1%	

*Clot lysis time was the dependent variable as measured by either turbidimetry or thromboelastography, yielding two different equations

Abbreviations: TAFI-thrombin activatable fibrinolysis inhibitor; PAI-plasminogen activator inhibitor; BMI-body mass index.

## Discussion

We report the novel finding that the *in-vitro* CLT predicts risk of hemorrhage and clinical response to plasminogen activator treatment for intermediate-risk PE. Patients with PE and a short TEG CLT who were treated with tenecteplase had an increased risk of hemorrhage, and patients with a prolonged TEG CLT had less benefit in terms of quality of life and exercise tolerance at three months. The TEG technique of assessing CLT appeared better suited at unmasking underlying resistance to lysis than the turbidimetric technique using light spectroscopy. For example, 18% of PE patients had a marked resistance to lysis, evidenced by a TEG CLT > 180 min (10,800 seconds), and the longest CLT from turbidimetry was 9,975 seconds. In multivariate analysis, the PAI-1 and TAFI protein concentrations were the most robust explanatory variables for a prolonged CLT with both techniques.

These findings have relevance in clinical settings where providers have administered a plasminogen-activating agent to patients with acute, intermediate-risk PE and subsequent complications of severe hemorrhage developed. Indeed, recent meta-analyses further do not support the use of thrombolytics in hemodynamically stable patients with acute PE [4–7]. These adverse outcomes increase the risk of morbidity and mortality, may lengthen hospital length of stay, and confound clinical decisions on appropriateness of fibrinolytic treatment of intermediate-risk PE. Thus, a biomarker to optimize the therapeutic index by personalizing the dose of plasminogen activating enzyme to the patient’s plasmatic resistance profile offers the potential to bridge current knowledge gaps in identifying which patients with intermediate-risk PE may be the best candidates for fibrinolytic therapy.

Our CLT and biomarker findings add to prior work showing that unrestrained fibrinolysis predicts hemorrhage risk and impaired fibrinolysis worsens treatment efficacy for VTE. Lobo et al., demonstrated elevated D-dimer levels, ostensibly a biomarker of hyperfibrinolysis, were associated with an increased rate of major bleeding [25]. In this registry study, 3 of the 8 patients with major or near major hemorrhages following tenecteplase treatment had elevated D-dimer levels in the fourth quartile. Conversely, prior work has established that hypofibrinolysis increases the risk of primary and recurrent venous thromboembolism. [26–32] This effect may contribute to a reduced long-term therapeutic effect of plasminogen activators, assuming that VTE recurrence worsens quality of life. In the current study, we demonstrate for the first time, to our knowledge, that a substantial percentage of patients with PE have a markedly prolonged TEG CLT at the time of treatment (and prior to the initiation of any fibrinolytics).

A variety of plasma-based clot lysis assays have been used to access CLT, including those using human thrombin together with tPA [33, 34], as well as those based on serial measurements of D-dimer release [35, 36]. The advantage of these methods to determine CLT is thought to derive overall plasma fibrinolytic capacity by representing the integrated effect of both procoagulant and profibrinolytic factors. The technique of turbidimetry (A405) under non-shear conditions has been used extensively in the past to assess global efficiency of lysis. The addition of an automated method to produce shear stress while measuring tPA-catalyzed CLT in plasma has only recently been described [37, 38].

To provide rigorous assessment, we measured CLT in all subjects using two methods. Overall, we found these two methods demonstrate good correlation and agreement in healthy subjects and also both identify a prolonged CLT in patients with metabolic syndrome and diabetes mellitus. Nevertheless, TEG was superior in identifying patients with intermediate-risk PE who exhibited a prolonged CLT (36.8% using TEG method versus 10% using the turbidimetry method). This implies a notable difference when measuring CLT under shear versus non-shear conditions for patients diagnosed with intermediate-risk PE and suggests that TEG may be a more sensitive technique for identifying abnormal CLT in these (and potentially other) patients. While we had no prior assumption that one method would be superior to the other, we postulate based on our findings that the mechanical action of TEG more closely mimics the *in-vivo* flow dynamics surrounding a pulmonary thrombus. With both methods, we corrected for the anticoagulant effect of therapeutic heparin, by adding excess lyophilized heparinase (sufficient to overcome 6IU/mL) prior to measurement of CLT. Additionally, we used Alteplase over Tenecteplase because Alteplase has FDA clearance to treat PE, and remains more widely available than Tenecteplase. Although Tenecteplase has a lower rate of PAI-1 inhibition than Alteplase (1.5x10^5^ M^-1^sec^-1^ versus 1.5x10^7^ M^-1^sec^-1^) the rate of PAI-1 neutralization is the primary determinant of the net catalytic activity in-vivo for Tenecteplase owing to its decreased clearance by the liver via the mannose receptor [39, 40]. The inhibitory action of TAFI affects plasmin binding to fibrin and is therefore independent of the plasminogen activator structure. For these reasons, we believe that PAI-1 and TAFI will remain accurate biomarkers of hemorrhage risk for patients treated with Alteplase or Tenecteplase. It is unclear at this point however, if screening plasmatic levels of PAI-1 and TAFI in hemodynamically stable patients with acute PE will move the benefit balance in favor of thrombolysis.

We observed that, 18.4% (n = 14) of patients from the TOPCOAT group, 50% (n = 5) of the metabolic syndrome group, and 40% (n = 4) of the diabetes mellitus group demonstrated increasing tensile clot strength over time. We hypothesize that this may be the result of tPA-induced release of fibrinopeptides A and B from fibrinogen that binds and protects soluble thrombin from inhibition, further perpetuating a pro-coagulant state [41, 42]. However, the concentration of tPA used in our experiments was much lower than that used in the prior experiments examining fibrinopeptide release.

Limitations include the fact that these data have no radiological evidence from TOPCOAT that definitively show evidence of resistance of pulmonary emboli to fibrinolysis. In addition to the number of samples being limited by the small number of patients with this pathology who met inclusion criteria in the original study, ten plasma samples from the original TOPCOAT trial were not analyzed due to: inability to draw blood (n = 1), patient withdrawal (n = 2), sample loss or no follow-up (n = 7). This small sampling of patients certainly limits the conclusions we may draw, but nonetheless maintains the work as hypothesis generating. Due to limited patient plasma supply, the TEG samples were run in singlet. The PAI-1 assay only measured total protein, as opposed to active enzyme, and it is possible that measurements of active enzyme could increase the importance of PAI-1 in the multivariate model. TOPCOAT samples had higher average BMI when compared to healthy controls (p = 0.041), a relevant point in view of a multivariate analysis that found obesity as the strongest predictor of quality of life in the TOPCOAT patients [43]. In the present work, analysis of variance, conducted to examine pairwise comparisons of BMI categories (overweight, obese, morbidly obese) with CLT by either turbidimetry or TEG, yielded no significant pattern of difference that would justify BMI as a confounding variable in CLT.

In conclusion, we found that the CLT is a highly variable phenotype that predicts the therapeutic index of fibrinolysis for intermediate-risk PE. The primary mediators of CLT are the plasma proteins PAI-1 and TAFI.

## Supporting Information

S1 Fig **A-D.** Measurement of clot lysis time (CLT) using turbidimetry (A) and TEG (B). Characteristic curves demonstrating normal (solid line), and resistance to fibrinolysis (dotted line) using turbidimetry (C) and TEG (D).
(DOCX)

S2 Fig Comparison of two methods of assessing resistance to fibrinolysis initiated by recombinant tissue plasminogen activator. (A) First order regression and (B) The Bland Altman plot (95% limits of agreement -1606 to +1921 seconds).
(DOCX)
