# Supplementary material for: Variable Resistance to Plasminogen Activator Initiated Fibrinolysis for Intermediate-Risk Pulmonary Embolism
Source: PLoS One. 2016 Feb 11;11(2):e0148747. doi: 10.1371/journal.pone.0148747 (PMC4751085; doi:10.1371/journal.pone.0148747)
Supplement: S1 Fig — A-D. Measurement of clot lysis time (CLT) using turbidimetry (A) and TEG (B). Characteristic curves demonstrating normal (solid line), and resistance to fibrinolysis (dotted line) using turbidimetry (C) and TEG (D). (DOCX) [file pone.0148747.s001.docx]

**Supplemental Figure 1A-D. Measurement of clot lysis time (CLT) using turbidimetry (A) and TEG (B). Characteristic curves demonstrating normal (solid line), and resistance to fibrinolysis (dotted line) using turbidimetry (C) and TEG (D).**

**
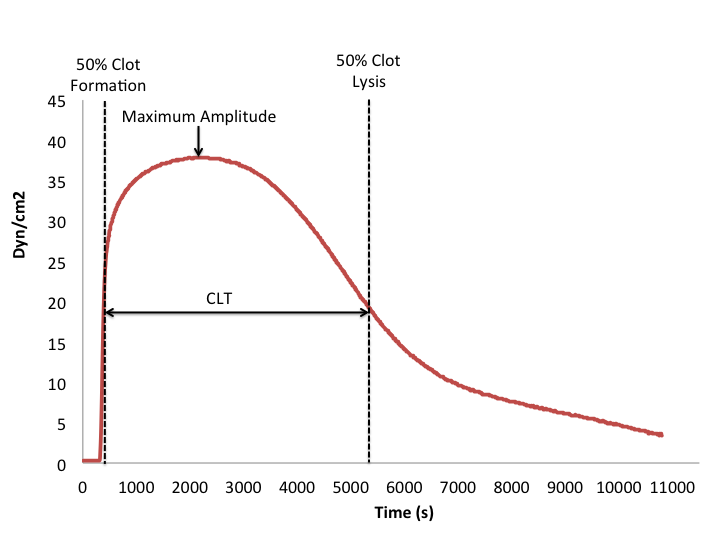

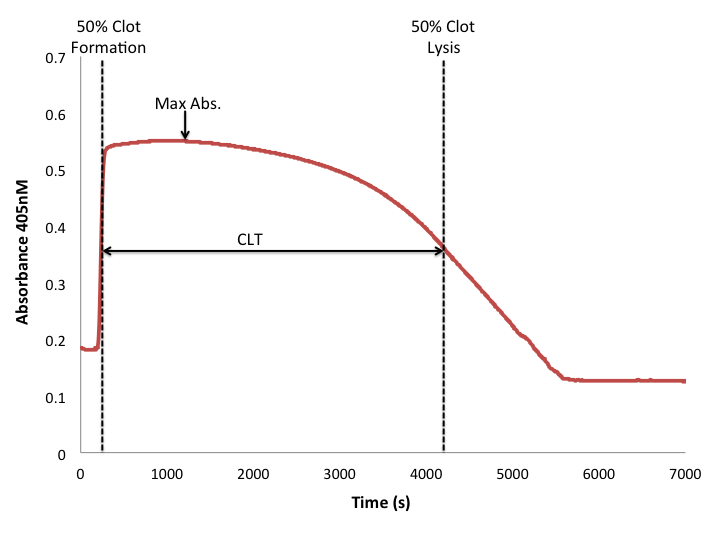
**

**A B**

**
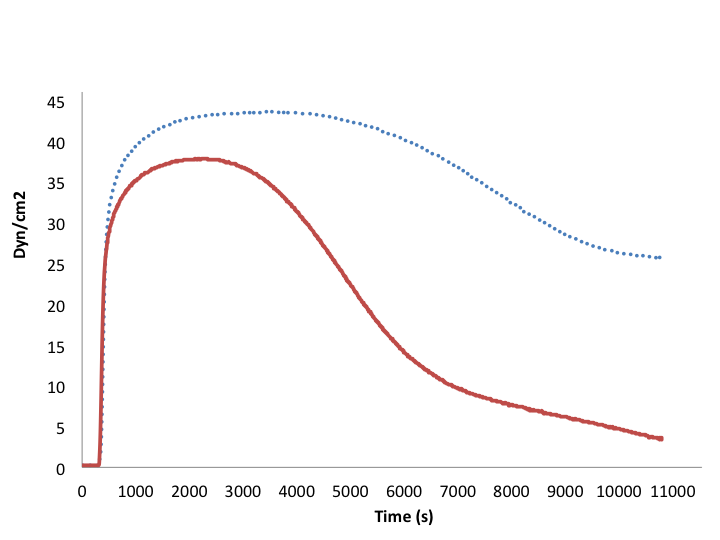
**

**
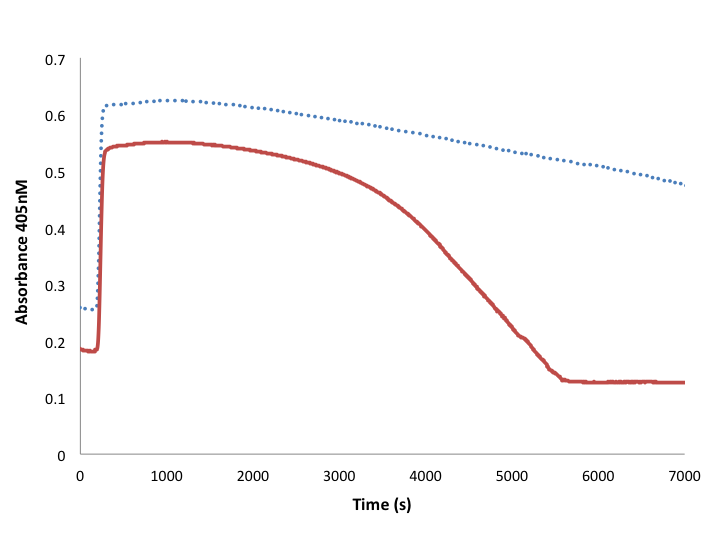
C D**
