# Supplementary material for: Variable Resistance to Plasminogen Activator Initiated Fibrinolysis for Intermediate-Risk Pulmonary Embolism
Source: PLoS One. 2016 Feb 11;11(2):e0148747. doi: 10.1371/journal.pone.0148747 (PMC4751085; doi:10.1371/journal.pone.0148747)

**Supplemental Figure 2. Comparison of two methods of assessing resistance to fibrinolysis initiated by recombinant tissue plasminogen activator. (A) First order regression and (B) The Bland Altman plot (95% limits of agreement -1606 to +1921 seconds).**


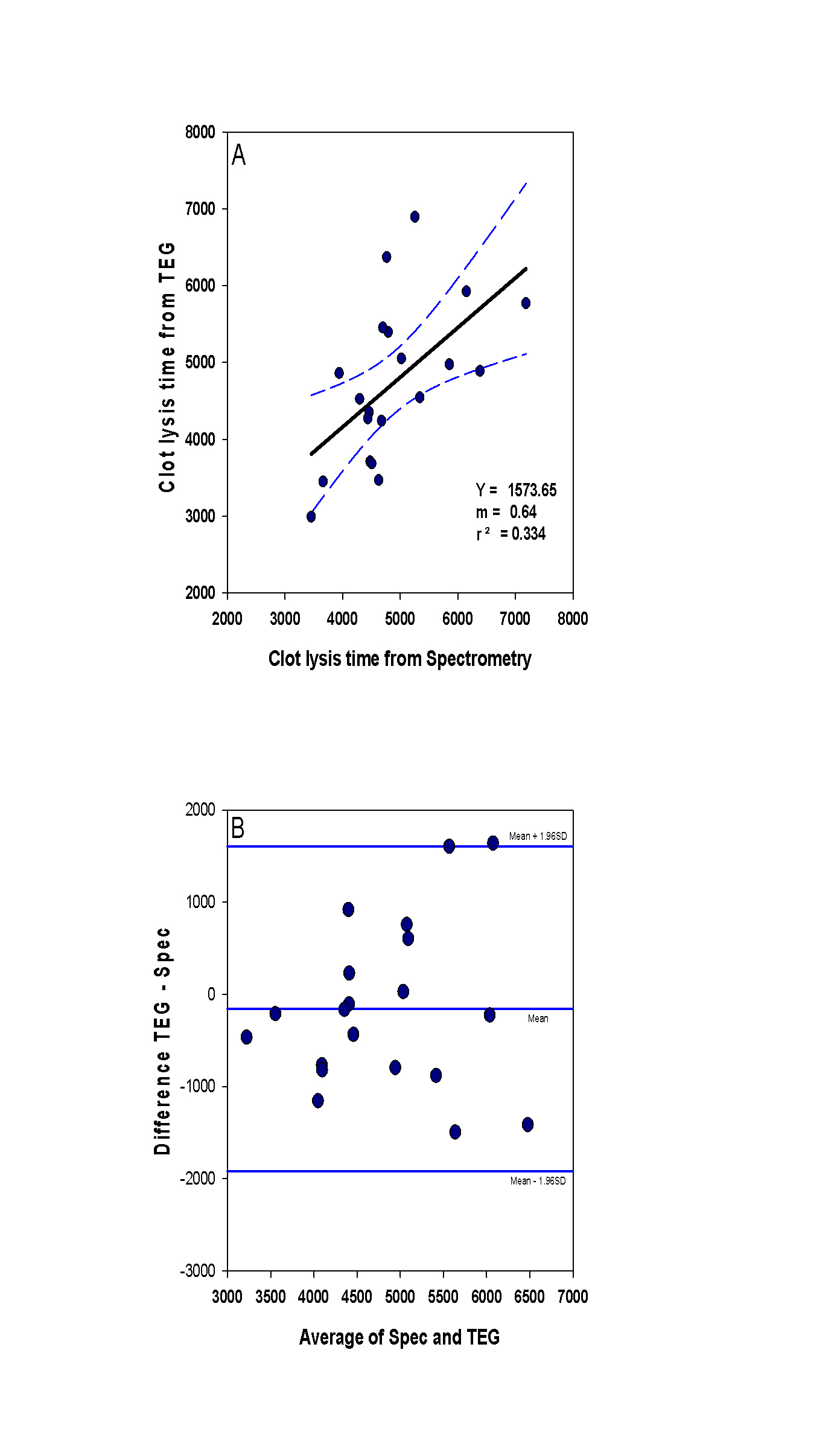

Supplement: S2 Fig — (A) First order regression and (B) The Bland Altman plot (95% limits of agreement -1606 to +1921 seconds). (DOCX) [file pone.0148747.s002.docx]
